# Positive selection screen identifies natural product β-catenin inactivators

**DOI:** 10.1073/pnas.2625189123

**Published:** 2026-09-25

**Authors:** Matthew W. Boudreau, Vitor F. Freire, Sophie C. Corbett, Lucero Martínez-Fructuoso, Shilpa R. Shenoy, Wenyu Yu, Rohitesh Kumar, Christopher C. Thornburg, Rhone K. Akee, Brian D. Peyser, Qinqin Jiang, Jennifer Splaine, Jamie L. Pfaff, Dongli Yu, Benjamin C. Chandler, Dinah M. Abeja, Katherine A. Donovan, Jianwei Che, Benjamin L. Lampson, Mariana Cooke, Marcelo G. Kazanietz, Patricia Szajner, Jennifer A. Smith, Vidyasagar Koduri, Tanja Grkovic, Barry R. O’Keefe, William G. Kaelin

**Affiliations:** ^a^ Department of Medical Oncology, Dana-Farber Cancer Institute and Harvard Medical School, Boston, MA 02215; ^b^ Broad Institute of Massachusetts Institute of Technology and Harvard, Cambridge, MA 02142; ^c^Natural Products Branch, Developmental Therapeutics Program, Division of Cancer Treatment and Diagnosis, National Cancer Institute, Frederick, MD 21701; ^d^Molecular Targets Program, Center for Cancer Research, National Cancer Institute, Frederick, MD 21701; ^e^ Natural Products Support Group, Leidos Biomedical Research, Inc., Frederick National Laboratory for Cancer Research, Frederick, MD 21701; ^f^Institute of Chemistry and Cell Biology-Longwood (ICCB-Longwood) Screening Facility, Harvard Medical School, Boston, MA 02115; ^g^ Department of Cancer Biology, Dana-Farber Cancer Institute, Boston, MA 02215; ^h^ Department of Biological Chemistry and Molecular Pharmacology, Dana-Farber Cancer Institute, Boston, MA 02215; ^i^ Department of Molecular and Cellular Pharmacology, University of Miami Miller School of Medicine, Miami, FL 33136; ^j^ Sylvester Comprehensive Cancer Center, University of Miami, Miami, FL 33136; ^k^Department of Immunology, Harvard Medical School, Boston, MA 02215; ^l^ Division of Hematology, Brigham and Women’s Hospital and Harvard Medical School, Boston, MA 02215; ^m^HHMI, Chevy Chase, MD 20815

**Keywords:** natural products, chemical biology, phenotypic screening, β-catenin

## Abstract

Many genetically validated targets in cancer, including the transcription factor β-catenin (β-cat), have historically been viewed as undruggable. Cell-based phenotypic screening of chemical compounds can reveal unanticipated biological and pharmacological principles. Natural products are powerful probes because of their superior structural diversity, drug-like properties, and biological activities as compared to unoptimized synthetic compounds. We screened 326,304 natural product mixtures (40,744 extracts and 285,560 fractions derived from them) using mammalian cells expressing an oncogenic version of β-cat fused to a suicide protein. Multiple fractions degraded the β-cat fusion protein or drove it into a compartment where both fusion partners were apparently inactive. The active natural product from one of the latter specifically activates novel, but not classical, protein kinase Cs and thereby relocates β-cat to juxtamembrane vacuolar structures. These findings suggest a path for inactivating oncogenic β-cat and underscore the power of screening natural product collections with robust phenotypic assays.

## Significance

Finding compounds that can inhibit genetically validated, but difficult to drug, targets is a pressing problem in drug discovery. Natural products often exhibit unexpected pharmacological mechanisms of action that are not easily predicted. Herein, for proof of concept, we sought natural products that can inactivate β-catenin (β-cat), which is a historically challenging cancer target. Utilizing a positive selection assay that fuses oncogenic β-cat to a suicide protein, we identified a natural product that activates novel-type protein kinase Cs (PKCs) and thereby mislocalizes and inactivates β-cat. This work illustrates the opportunity of combining so-called “up-assays” and complex natural product mixtures to discover active natural products that define new mechanistic paradigms to combat challenging target proteins.

Many genetically validated intracellular targets for various diseases are viewed as difficult to tackle with drug-like molecules because they are believed to lack appropriate hydrophobic pockets (1, 2). Examples of such targets in cancer include oncogenic versions of K-Ras, c-Myc, and β-cat, although there has been significant progress drugging K-Ras with two FDA approved inhibitors for K-RAS G12C (3) and impressive clinical efficacy of RAS(ON) tricomplex inhibitors (4). The discovery that thalidomide-like drugs (“IMiDs”) are “molecular glues” that reprogram the cereblon ubiquitin ligase to target two otherwise undruggable oncogenic transcription factors, IKZF1 and IKZF3, for degradation (5, 6) has spurred interest in identifying additional molecules that can degrade specific target proteins by hijacking particular ubiquitin ligases.

There are many other ways, however, that a small molecule could downregulate a protein of interest (POI). To search for degraders in a mechanism-agnostic fashion, we previously created a cell-based degrader positive selection assay (i.e., an “up assay”) that uses a bicistronic reporter encoding: 1) the POI fused to a modified deoxycytidine kinase (DCK*) that converts the non-natural nucleoside BVdU to a toxin and 2) green fluorescent protein (GFP) (7). GFP facilitates sorting for cells with the desired levels of the fusion protein and the quantification of cells that are both viable and retain the reporter. Perturbants that lower the abundance of the POI (and hence the POI fusion) promote the survival of GFP-positive viable cells in the presence of BVdU (7–9). “Up assays” are less likely than “down assays” to yield trivial positives that simply interfere with cellular housekeeping functions or are otherwise toxic (10).

Mutations that cause the accumulation of active β-cat, including inactivating mutations of the *APC* or *AXIN* genes and activating mutations of *CTNNB1,* which encodes β-cat, are common in many cancers, including colon, gastric, liver, and uterine cancers (11–13). β-cat functions in the Wnt pathway, which plays important roles in stem cell biology, development, and cancer (12). A first-in-class β-cat inhibitor that blocks β-cat binding to its partner TCF4 recently entered clinical trials (14). Other β-cat inhibitors, including putative degraders, have been described, but they do not appear to be robust (15).

## Results

**Fig. 1.**
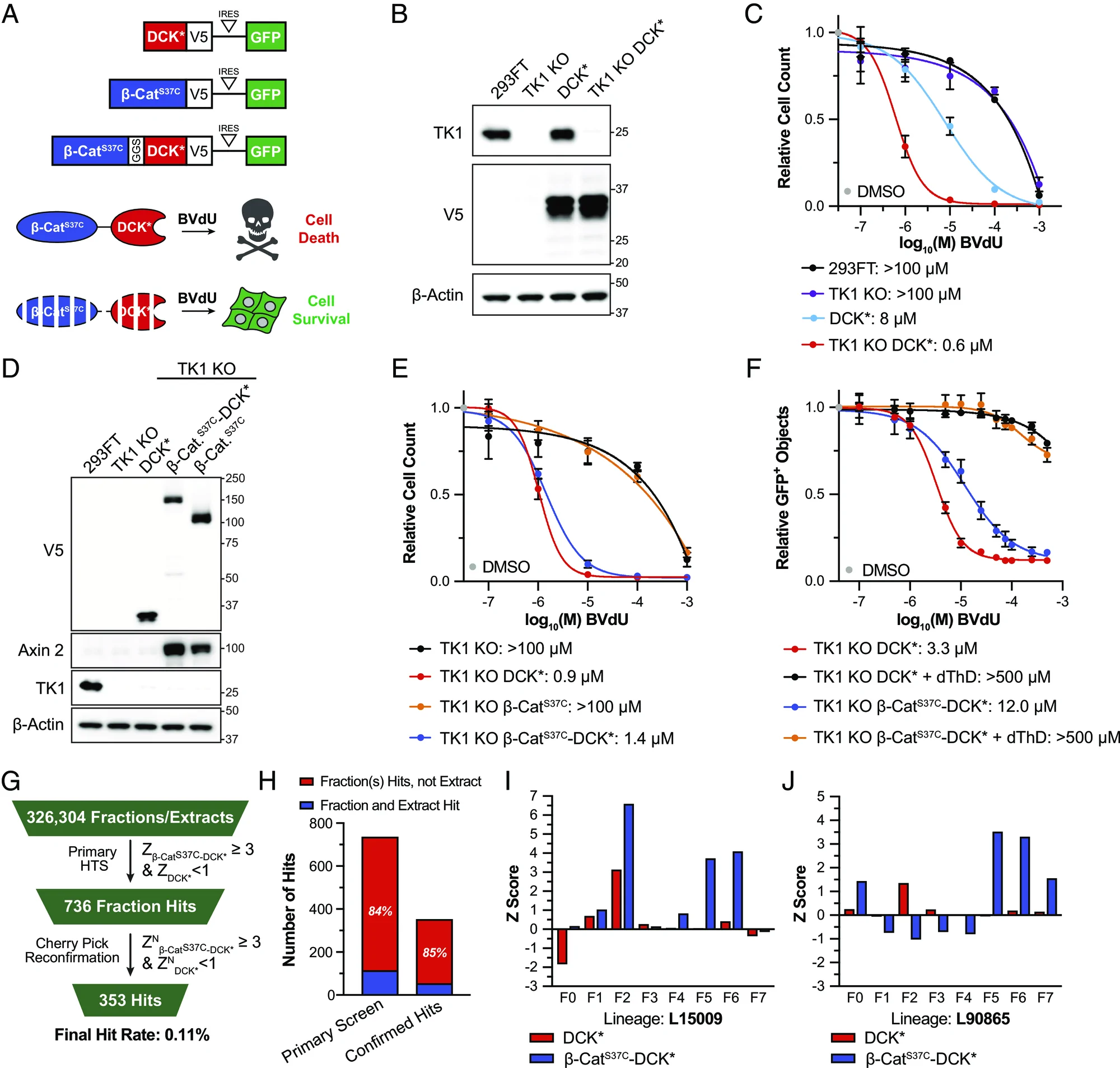
Establishment of β-Cat^S37C^-DCK* positive selection system for natural product screening. (*A*) Reporter schematics and representation of the positive selection assay for β-Cat^S37C^ degradation. DCK*: variant deoxycytidine kinase with S74E/R104M/D133A substitutions. GGS: Gly-Gly-Ser spacer. IRES: internal ribosomal entry site. (*B*) Immunoblot analysis of 293FT cells expressing DCK* with or without TK1 inactivated using CRISPR/Cas9 (“TK1 KO”). (*C*) Relative survival of 293FT cells shown in (*B*) treated with the indicated concentrations of BVdU for 96 h. n = 3 biological replicates. (*D*) Immunoblot analysis of 293FT cells expressing the reporters shown in (*A*). (*E*) Relative survival of 293FT cells depicted in (*D*) treated with the indicated concentrations of BVdU for 96 h. n = 3 biological replicates. (*F*) Relative GFP+ objects for 293FT cells depicted in (*D*) treated with DMSO or dThD (final concentration 100 µM) for 24 h followed by the indicated concentrations of BVdU for 96 h in 384-well format. n = 3 technical replicates. (*G*) Summary of high-throughput screening of natural product extract and fraction library and the hit scoring metrics used. Z: Z-score based on variation among experimental wells. Z^N^: Z-score based on the variation among the negative (^N^) control wells. (*H*) Number of positively scoring fractions for which corresponding extract did (blue) or did not (red) also score. (*I* and *J*) Z-scores from the primary screen of the L15009 lineage (*I*) and L90865 lineage (*J*).

To search for novel β-cat degraders, we engineered 293FT cells (which are β-cat-independent) to express an oncogenic β-cat variant (S37C) fused to DCK* (β-cat^S37C^-DCK*) or, as controls, unfused β-cat S37C (β-cat^S37C^) or unfused DCK* (Fig. 1*A*). In earlier whole genome CRISPR screens (8, 9, 16) we noted that inactivation of thymidine kinase 1 (TK1) sensitized DCK*-positive cells to BVdU, presumably because TK1 monophosphorylates thymidine, which competes with BVdU, without shifting the BVdU sensitivity of DCK*-negative cells. We therefore inactivated TK1 in the 293FT cells using CRISPR/Cas9 knockout prior to introducing the different reporters (Fig. 1 *B* and *C*). β-cat^S37C^-DCK*, like β-cat^S37C^, increased Axin 2 levels, a well-established β-cat/Wnt target gene, suggesting that the β-cat moiety in the β-cat^S37C^-DCK* fusion is properly folded and functional (Fig. 1*D*).

As expected, the BVdU IC_50_ values for 293FT TK1 KO cells expressing β-cat^S37C^-DCK* or unfused DCK* were 2 to 3 logs lower than for the parental 293FT TK1 KO cells or 293FT TK1 KO cells expressing unfused β-cat^S37C^ (Fig. 1*E*). The sensitivity of 293FT TK1 KO cells expressing unfused DCK* to BVdU was not altered by cotreatment with the β-cat stabilizer CHIR99021 (*SI Appendix*, Fig. S1 *A* and *B*), which disrupts a β-cat phosphodegron by inhibiting the GSK3 kinase, or by coexpression of unfused β-cat^S37C^ (*SI Appendix*, Fig. S1 *C* and *D*). Therefore, β-cat signaling does not, per se, alter killing by BVdU in the presence of DCK*.

In pilot experiments, we confirmed that CRISPR sgRNAs directed against DCK* and, to a lesser extent, against β-cat, promoted the survival of the 293FT TK1 KO cells expressing β-cat^S37C^-DCK* (*SI Appendix*, Fig. S1 *E*–*G*). As expected (7), killing of 293FT TK1 KO cells expressing β-cat^S37C^-DCK* or unfused DCK* was reversed by the addition of 100 μM thymidine (dThD) in low- throughput assays and in 384-well plate format (Fig. 1*F*). The Z-prime (Z’) factors for the latter were >0.5 using the assay positive dThD as a surrogate for a specific true positive (*SI Appendix*, Fig. S1 *H* and *I*). Consistent with our previous experience with other DCK* fusions, multiple compounds that downregulate proteins by nonspecifically interfering with transcription (e.g., actinomycin D), translation (e.g., zotatifin), or protein folding (HSP90 inhibitor: 17-AAG) did not score as hits in the β-cat^S37C^-DCK* reporter cells (*SI Appendix*, Fig. S1*J*). BIX-02565, which inhibits translation by inhibiting RSK2, protected both β-cat^S37C^-DCK* and unfused DCK* cells (*SI Appendix*, Fig. S1*J*), although the significance of this is unclear and its effects in this assay might be off-target.

Encouraged by these findings, we next conducted a pilot high-throughput screen (HTS) using curated commercial and academic collections of bioactive compounds with known mechanisms of action (*Materials and Methods*) (a total of 2,699 compounds) against the β-cat^S37C^-DCK* and DCK* reporter cells grown in 384-well plates (*SI Appendix*, Fig. S2*A*). Experimental compounds were added to the assay plates using acoustic dispensing (day 0). BVdU was added on day one and GFP+ objects per well were measured on day five using a laser scanning imaging cytometer and the values were converted to Z-scores. Eleven of the compounds scored in the β-cat^S37C^-DCK* cells and not in the unfused DCK* cells (Z ≥ 3 β-cat^S37C^-DCK*; Z < 1 for DCK*) (*SI Appendix*, Fig. S2*A*). Five of the 11 were closely related to compounds that scored in cells expressing unrelated DCK* fusions in our experience and were therefore not studied further.

Of the remaining six compounds, one, **AZ-628**, downregulated both exogenous β-cat^S37C^-DCK* and endogenous β-cat^WT^ (*SI Appendix*, Fig. S2*B*), suggesting it was a true positive. Intriguingly, **AZ-628** is a BRAF targeting, type 2 kinase inhibitor (17) that structurally resembles a compound dubbed WNTinib that inhibits downstream Wnt signaling, at least in part, by blocking the phosphorylation of EZH2 (18). WNTinib did not score in our assay and did not affect β-cat levels (*SI Appendix*, Fig. S2 *C* and *D*). We did not pursue **AZ-628** further because sensitivity to **AZ-628** does not track with β-cat dependence (in contrast with the good correlation with BRAF dependency) in public databases (DepMap Drug Repurposing Database, *SI Appendix*, Fig. S2 *E* and *F*) (19, 20) and **AZ-628** was highly toxic at concentrations just above those used in our screen. These observations might reflect its known polypharmacology. Nonetheless, **AZ-628** might eventually illuminate a tractable path for degrading β-cat and, if so, could be a useful compound for additional studies.

The compounds found in nature are far more structurally diverse than synthetic chemicals. Screening natural product mixtures is challenging, however, for multiple reasons (21). For example, toxic compounds in such mixtures can cause false positives in “down” assays and false negatives in “up” assays (10). Moreover, it can be difficult to isolate and identify the active principle compound(s) from complex natural product mixtures when those mixtures score positively in a screen (21). We reasoned that both these challenges could be partially mitigated by marrying the performance characteristics of our screen to the use of partially fractionated natural product mixtures. Prefractioned natural product libraries offer many advantages, including sequestering toxic and nuisance compounds, concentrating minor active compounds, and simplifying downstream chemistry efforts (22, 23). The National Cancer Institute (NCI) Program for Natural Product Discovery (NPNPD) has prefractionated natural product organic extracts from the NCI natural product repository using a C8 solid phase extraction column into seven fractions (F1-F7) (22). Each unfractionated extract (F0), which is designated with a letter code based on its source (“L” = terrestrial plant; “M” = marine; H = fungal; “K” = marine plant) and a 5-digit unique identifier, is screened together with its seven subfractions (F1-F7) (e.g., “L90865_5” is the fifth fraction, F5, from the terrestrial plant extract L90865). We refer to each extract and its progeny as a lineage.

As an early risk assessment experiment, we screened two NPNPD challenge plates against β-cat^S37C^-DCK* and DCK* cells grown in 384-well plates as described above. These plates include extracts and fractions that either contain known pan-assay interference (PAIN) compounds or are highly pan-toxic in the NCI-60 assay. Our assay performed well, with excellent correlation between replicates and did not yield any positive hits (Z ≥ 3 β-cat^S37C^-DCK*; Z < 1 for DCK*, *SI Appendix*, Fig. S3*A*), suggesting that our hit rate moving forward would not be prohibitively high. Excellent replicate-to-replicate correlations (R^2^ = 0.96 to 0.98) continued as the HTS began with the first 10 plates of fractionated natural product samples [440 extracts (F0) and 3,080 fractions (F1-F7), *SI Appendix*, Fig. S3 *B* and *C*]. Thus, the remainder of the HTS campaign was conducted with a single replicate per plate for β-cat^S37C^-DCK* and DCK* cells.

We ultimately screened 326,304 samples (40,744 F0 and 285,560 F1-F7) (Fig. 1*G*). As expected, the Z’ factors were excellent throughout the screen, Z-scores for the two different reporter lines were highly correlated, and most of the samples did not affect BVdU killing in either cell line (Z ≈ 0, *SI Appendix*, Fig. S3 *D*–*F*). 736 samples corresponding to 695 lineages scored as potential β-cat degraders (Z ≥ 3 β-cat^S37C^-DCK*; Z < 1 for DCK*, *SI Appendix*, Fig. S3*E*). Excluded from these 736 hits were samples where the F0 scored, but none of the corresponding F1-F7 samples scored. 353 (48%) hits retested positively (here using the negative (N)-control based Z^N^ metric because of the presumed enrichment for true positives) after being cherry-picked and rescreened in duplicate, for a final hit rate of 0.11% (Fig. 1*G*).

The 353 hits, corresponding to 334 unique natural product lineages, originated from 70 different countries/regions (Dataset S1) and were derived from terrestrial plants (72.8%), marine sources (25.2%), marine plants (1.1%), and fungal/microbial sources (0.8%) (*SI Appendix*, Fig. S3*H*). These percentages broadly mirrored the composition of the screened samples (*SI Appendix*, Fig. S3*I*). Hits were derived from a range of taxonomies (Dataset S1).

Most of the hits came from the more lipophilic F5 and F6 fractions (*SI Appendix*, Fig. S3*J*), likely because scoring in our assay requires cell permeability. For >80% of the hit fractions, the crude extract (F0) did not likewise score as a hit (Fig. 1*H*), perhaps because the F0 contained a compound(s) that masked the activity in the scoring subfraction or due to increased concentration of minor active compounds after separation into the individual fractions. Unsurprisingly, given the single column separation at this point, we noted 16 lineages where adjacent fractions scored. Both these observations were exemplified by the analysis of L15009 and L90865 (Fig. 1 *I* and *J*).

Three hundred and twenty-two hit fractions were separated into 22 subfractions (sF1-sF22) by preparative reverse-phase HPLC (C18 column), yielding 7,084 subfractions (some hits were not subfractionated due to insufficient supply of the parent extract or other technical considerations) (*SI Appendix*, Fig. S4*A*). It is important to note that the exact concentration (µg/mL) for each subfraction was not known and was estimated from an equally distributed mass balance (*Materials and Methods*). Therefore, subfractions were screened, in duplicate, at two doses (100 nL and 200 nL). Subfraction hits were called positive if they scored at either or both doses (see hit metrics in *Materials and Methods*). 435 subfractions, derived from 181 lineages, scored positively and were then counterscreened against 293FT TK1 KO cells expressing DCK*-IKZF1. 398 subfractions, derived from 162 lineages, promoted the survival of β-cat^S37C^-DCK* cells, but not DCK*-IKZF1, and were studied further (*SI Appendix*, Fig. S4*A*). Scoring subfractions were well distributed across sF7-sF22 (*SI Appendix*, Fig. S4*B*), reflecting the lipophilic nature of most of their parental fractions (*SI Appendix*, Fig. S3*J*).

**Fig. 2.**
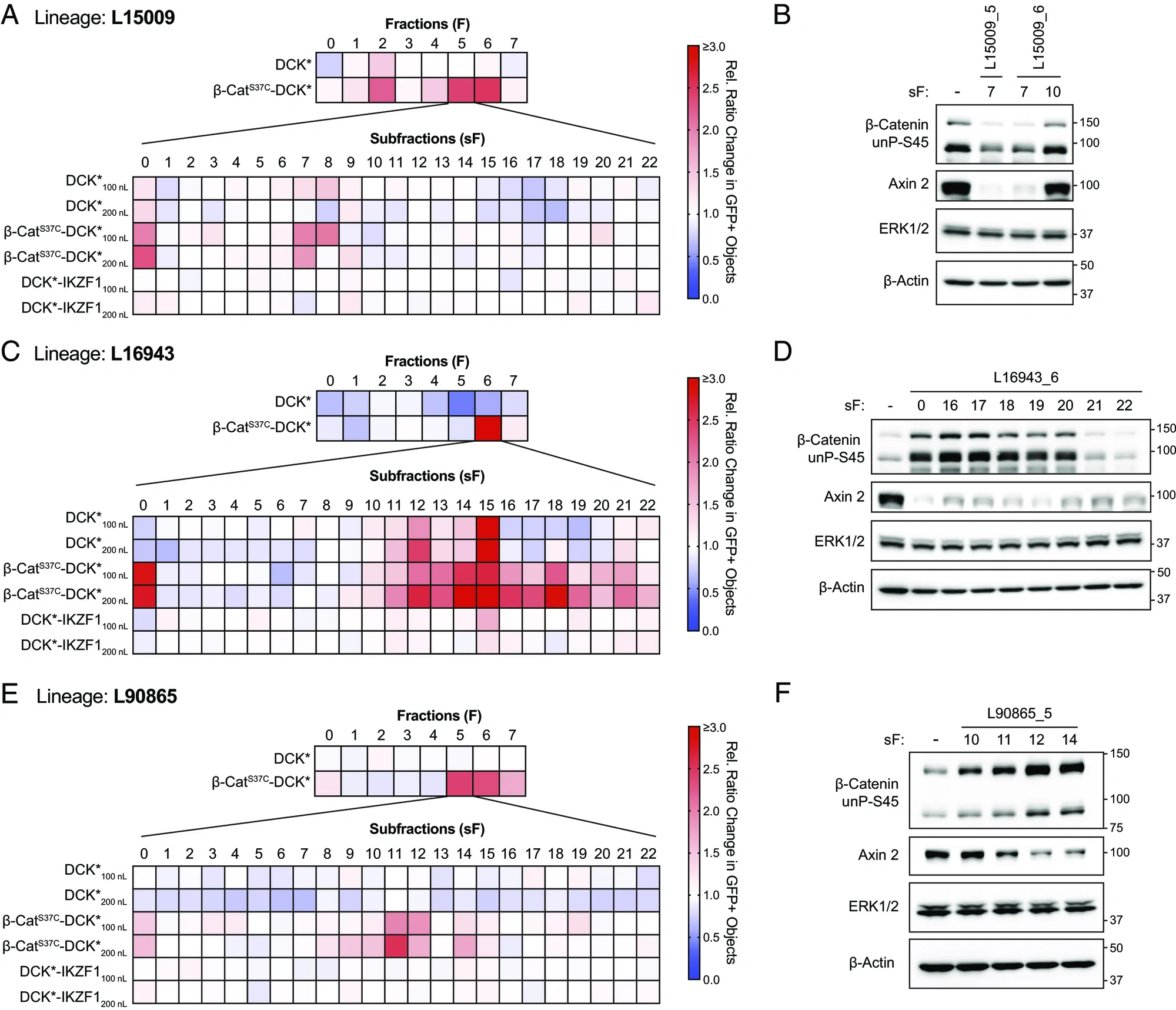
Two apparent modes of β-cat inactivation displayed by natural product subfractions. (*A*) Heatmap showing primary and secondary screening data, based on cell survival (GFP+ objects), related to lineage L15009 (fraction L15009_5, see fraction L15009_6 displayed in *SI Appendix*, Fig. S4*C*). (*B*) Immunoblot analysis of 293FT TK1 KO β-cat^S37C^-DCK* cells treated with the indicated subfractions from (A and *SI Appendix*, Fig. S4*C*) for 24 h. Note differential mobility of exogenous β-cat^S37C^-DCK* and endogenous β-cat. (*C*) Heatmap showing primary and secondary screening data related to lineage L16943. (*D*) Immunoblot analysis of 293FT TK1 KO β-cat^S37C^-DCK* cells treated with indicated subfractions from (*C*) for 24 h. (*E*) Heatmap showing primary and secondary screening data related to lineage L90865. (*F*) Immunoblot analysis of 293FT TK1 KO β-cat^S37C^-DCK* cells treated with indicated subfractions from (*E*) for 24 h. For all heatmaps, coloring is representative of the relative GFP+ objects measured in a given test well. 100 nL and 200 nL refers to the transfer volume for subfraction dosing. n = 2 technical replicates for all subfraction data and data are shown as an average. All immunoblot images are representative of two biological replicates. “-” = DMSO treatment.

Given the limited amount of material available, we immunoblotted β-cat^S37C^-DCK* cells at a single time point (24 h) and a single nominal concentration with at least one positive subfraction for each of the 162 extracts that scored positively. 36 lineages had at least one subfraction that modulated β-cat^S37C^-DCK* abundance *and* decreased the expression of the canonical β-cat target Axin 2. This included 24 extracts with subfractions that decreased β-cat^S37C^-DCK* abundance (hereafter referred to as “downregulators”) and, unexpectedly, 12 with subfractions that increased β-cat^S37C^-DCK* abundance (see examples of both in Fig. 2 and *SI Appendix*, Fig. S4*C*). It thus appeared that the latter inactivated the β-cat^S37C^-DCK* fusion protein, including both its β-cat and DCK* moieties, and thus protected cells against BVdU killing despite increasing exogenous β-cat^S37C^-DCK* levels (hereafter called “inactivators”).

We used our previously described workflow to further isolate pure/semipure compounds from the target lineages on a small, testable milligram scale (0.1 to 1 mg). This enabled preliminary structural annotation based on multiple spectroscopic analyses (24). Samples were first tested in the β-cat^S37C^-DCK* and DCK* BVdU killing assays. Selective positives were then tested for β-cat^S37C^-DCK* modulation in western blot assays to prioritize hits worth scaling up for complete structural elucidation and further in-depth exploration.

From the downregulator lineage L15009, we ultimately isolated a known neolignan as a mixture of jatrointelignan A and B epimers (**1**) (*SI Appendix*, Fig. S5*A*) (25). Compound **1** protected β-cat^S37C^-DCK* cells, but not DCK* cells, against BVdU treatment (*SI Appendix*, Fig. S5*B*). The epimeric mixture was unstable and difficult to isolate, leading to a lack of material for follow-up studies, including western blot studies of endogenous β-cat. Active subfractions from L15009 downregulated endogenous β-cat and Axin 2 in SNU398 cells (β-cat^S37C^ hepatocellular carcinoma cell line, *SI Appendix*, Fig. S5*C*). For the other downregulator lineages, either the pure compounds we isolated did not validate in secondary assays or we failed to arrive at a pure compound from the limited amount of extract available. The former could reflect false positives or failure to correctly isolate the active principle compound.

We then turned to the putative inactivators (Dataset S2). Most of these came from the same plant family, *Euphorbiaceae* (9 out of 12), suggesting that they contain the same or similar compounds responsible for the inactivator phenotype. Surprisingly, multiple inactivators increased the abundance of the DCK* fusion protein (or unfused DCK*) and, more variably, the coexpressed GFP reporter, when tested against a panel of DCK* fusion proteins, suggesting that they nonspecifically increase CMV promoter activity (*SI Appendix*, Fig. S6*A*). The differential effects between the DCK* fusions and GFP could reflect differences in protein half-life and cap-dependent versus cap-independent translation. Nonetheless, protection by the inactivators was always specific to the cells expressing full-length β-cat^S37C^ fused to DCK* (*SI Appendix*, Fig. S6 *B*–*D*). We discovered, using mass spectrometry proteomics of DLD-1 colorectal cancer cells, that a partially purified inactivator from L16943 induced JunB and other AP-1 family members (*SI Appendix*, Fig. S6*E*). JunB was robustly induced in cells treated with inactivators (*SI Appendix*, Fig. S6 *F* and *G*). Induction of AP-1 could explain the activation of the CMV promoter, but not the specific protection of full-length β-cat^S37C^ fused to DCK* (26–31).

Since the inactivators protected against BVdU killing despite increasing β-cat^S37C^-DCK* protein levels, we hypothesized that they aggregate or mislocalize β-cat such that β-cat (as well as the fusion protein) is functionally inactive. Treating DLD-1 cells, which have hyperactive β-cat due to an *APC* mutation, with active subfractions from L16943, as well as similar impure samples from L16945 (derived from the root bark of the same plant but not originally included in our screen) relocalized β-cat to what appeared to be large intracellular vacuoles abutting the cell membrane (*SI Appendix*, Fig. S7 *A* and *B*).

**Fig. 3.**
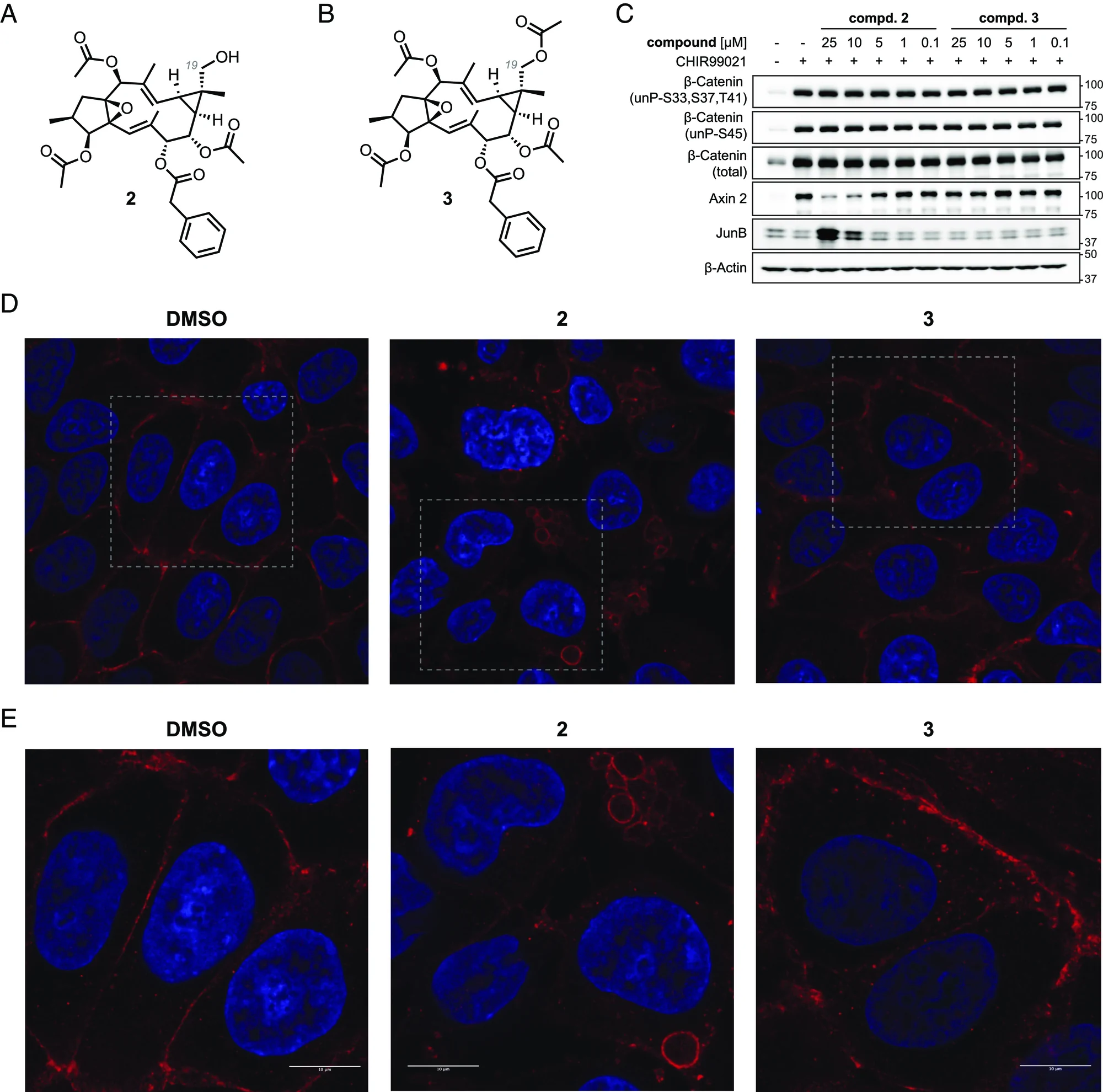
The active principle for inactivator lineage L90865 (compound **2**) inactivates and relocalizes β-Cat. (*A* and *B*) Chemical structures of compounds **2** and **3**. The only difference between **2** and **3** is the acetylation of the C19 (gray) alcohol. (*C*) Immunoblot analysis of 293FT cells treated with CHIR99021 (5 µM) and **2** or **3** at the indicated concentrations and incubated for 24 h. (*D* and *E*) Immunofluorescence images (W1: *D* or SORA enhanced: *E*) of DLD-1 cells treated with **2** or **3** at 25 µM concentration for 24 h. Blue: DAPI stain, Red: β-Cat. (Scale bar, 10 µm.)

During the purification of the L90865 inactivator subfractions, we isolated two highly related lathyrane natural products: one containing a free primary alcohol (**2**, also called **lathyranol**) and the other in which that primary alcohol is acetylated (**3**, also called **lathyranol-19-acetate**) (Fig. 3 *A* and *B*). Compound **2**’s primary alcohol is a unique substitution (i.e., on C19 of the geminal dimethylcyclopropane ring) for this class of natural products, which are rarely hydroxylated at this site (32, 33). Compound **2**, but not **3**, reduced β-cat activity, as determined by Axin 2 levels, in 293FT cells treated with the β-cat stabilizer CHIR99021, which inhibits GSK3, and mRNA expression of β-cat target genes *AXIN2*, *LEF1*, and *LGR5* in DLD-1 cells (Fig. 3*C* and *SI Appendix*, Fig. S7 *C* and *D*). The inactivators did not induce endogenous β-cat levels, in contrast to exogenous β-cat^S37C^-DCK*, presumably because the latter is driven by the CMV promoter (vide supra). Compound **2**, but not **3**, recapitulated the relocalization of β-cat described above (Fig. 3 *D* and *E* and *SI Appendix*, Fig. S7*E*). Of note, compound **2** was stable after 24-h incubation in cell media with no hydrolysis of acetyl groups observed (*SI Appendix*, Fig. S7*F*).

**Fig. 4.**
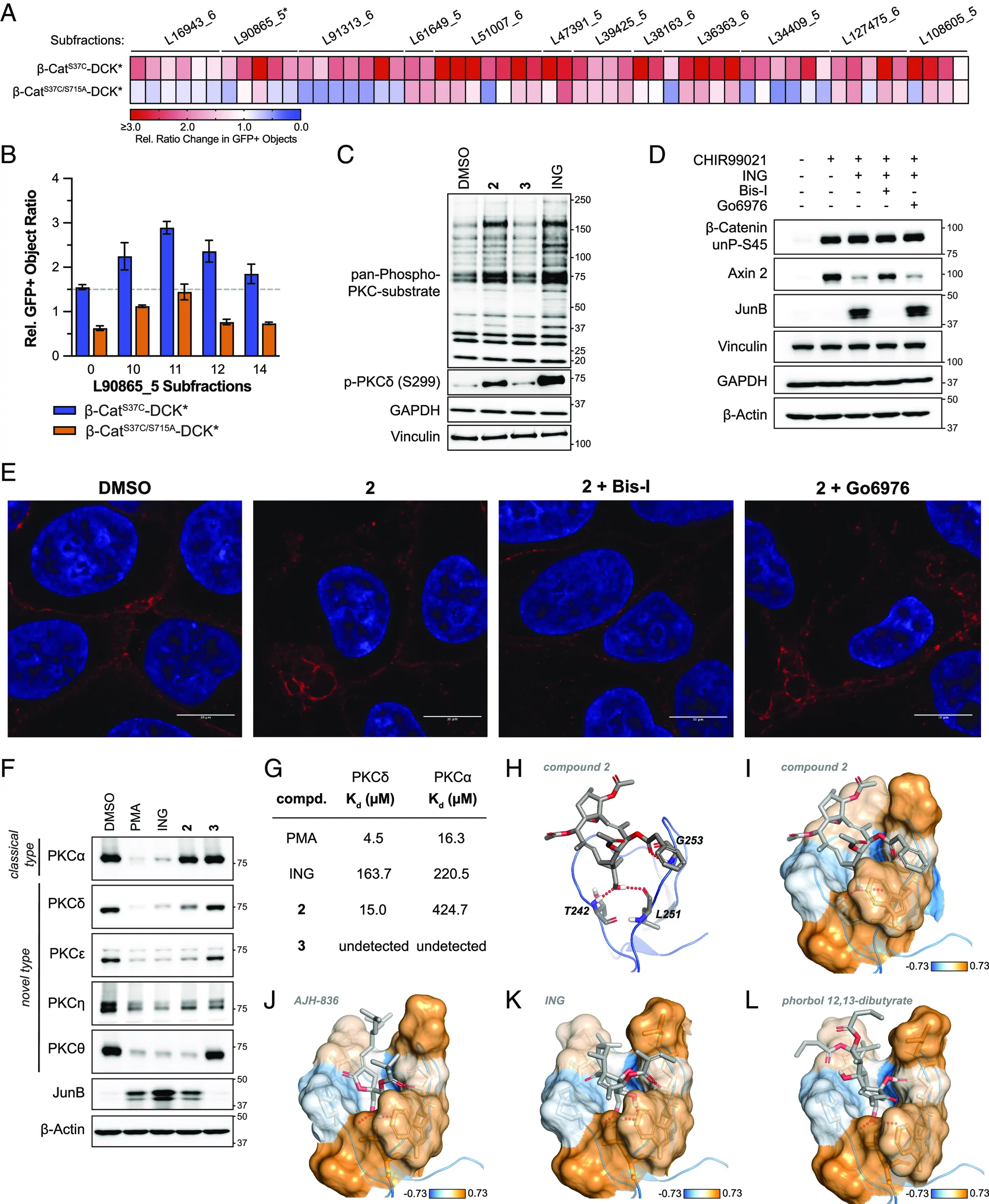
Compound **2** specifically activates novel PKC family members. (*A*) Heatmap showing cell survival data (GFP+ objects) for 293FT TK1 KO β-cat^S37C^-DCK* and 293FT TK1 KO β-cat^S37C/S715A^-DCK* cells treated with positive subfractions from the indicated lineages for 24 h followed by BVdU (50 µM) for 96 h. Subfractions were dosed at a nominal concentration of 2.5 µg/mL. *: L90865_5 series was dosed at 5 µg/mL. n = 2 technical replicates. (*B*) Relative GFP+ objects for 293FT TK1 KO β-cat^S37C^-DCK* and 293FT TK1 KO β-cat^S37C/S715A^-DCK* cells treated with L90865 subfractions for 24 h followed by BVdU treatment (50 µM) for 96 h. n = 2 technical replicates. (*C*) Immunoblot analysis of 293FT cells treated, where indicated, with **2** (25 µM), **3** (25 µM), or ING (1 µM) for 20 min. n = 3 biological replicates. (*D*) Immunoblot analysis of 293FT cells treated, where indicated, with CHIR99021 (5 µM), ING (1 µM), Bis-I (1 µM), or Go6976 (0.5 µM) for 24 h. n = 3 biological replicates. (*E*) Immunofluorescence of DLD-1 cells treated with indicated compounds **2** (25 µM), Bis-I (1 µM), or Go6976 (0.5 µM) for 24 h. Blue: DAPI stain, Red: β-Cat. (Scale bar, 10 µm.) (*F*) Immunoblot analysis of 293FT cells treated, where indicated, with PMA (1 µM), ING (1 µM), **2** (25 µM), or **3** (25 µM) for 24 h. n = 2 biological replicates. (*G*) Summary of the K_d_ values measured by microscale thermophoresis for each of the indicated compounds in the presence of full-length protein PKCδ or PKCα. (*H*) Three-dimensional representation of the molecular docking of compound **2** to the C1b domain of PKCδ (PDB: 7LF3). Key hydrogen bonds are shown with red dots. (*I*–*L*) Space-filled models for the simulated binding of compound **2** (*I*) and the reported crystal structures for AJH-836 (*J*, PDB: 7LF3), ING (*K*, PDB: 7KO6), and phorbol 12,13-dibutyrate (*L*, PDB: 7KNJ). Key hydrogen bonds are shown with red dots, and the coloring is based on the Eisenberg consensus hydrophobicity scale (note: a value less than −0.73 is set to −0.73).

The structure of compound **2**, together with the requirement for its primary alcohol, led us to ask whether it was a PKC activator, especially since: 1) PKC activators have been isolated from *Euphorbiaceae* before (32), 2) other lathyrane natural products resembling compound **2** can activate PKCs (34), 3) PKC activation, via ERK and JNK, increases JunB transcription and stability (28, 35), 4) PKC activation can increase CMV promoter activity via AP-1 (including JunB) and cap-dependent translation via 4E-BP1 (26–31), 5) PKC activation has been reported to cause similar vacuolization (also referred to as “budding”) of β-cat (36, 37), and 6) PKC can phosphorylate β-cat on Serine 715 (38, 39), which is removed by the C-terminal truncation that abrogates protection by the inactivators we tested (*SI Appendix*, Fig. S6 *A*–*D*). Regarding the latter, we discovered that the S715A mutation substantially reduced the BVdU protection by 9 of the 12 inactivator lineages, including L90865 extract, fractions, and subfractions that produced compound **2** (Fig. 4 *A* and *B* and *SI Appendix*, Fig. S8*A*). The S715A mutation did not, itself, affect reporter expression nor BVdU sensitivity (*SI Appendix*, Fig. S8 *B* and *C*). Moreover, compound **2** and the panPKC agonist ingenol-3-angelate (ING), but not compound **3**, induced the phosphorylation of multiple PKC substrates within 20 min of addition to cells (Fig. 4*C*). This coincided with phosphorylation of PKCδ on a site indicative of PKCδ activation (40, 41) (Fig. 4*C*). In further support of a role for PKC, both ING and the panPKC activator phorbol-12-myristate (PMA), phenocopied compound **2** with respect to the induction of β-cat^S37C^-DCK*, induction of JunB, and downregulation of Axin 2 in the 293FT TK1 KO β-cat^S37C^-DCK* cells (*SI Appendix*, Fig. S8*D*). They also phenocopied compound **2** with respect to endogenous β-cat relocalization and target gene expression in DLD-1 cells (*SI Appendix*, Figs. S7*C* and S8*E*). Relocalization of β-cat by ING occurred within 2-4 h of treatment (*SI Appendix*, Fig. S8*F*).

RNA sequencing (RNAseq) analysis of DLD-1 cells treated with PMA, ING, or compound **2** exhibited similar changes in gene expression (*SI Appendix*, Fig. S8 *G* and *H*). As expected with PKC activators, many genes were up- or downregulated. These changes were specific because the negative control compound **3** caused minimal changes in gene expression (*SI Appendix*, Fig. S8*I*). Gene-set enrichment analysis for MSigDB hallmark terms showed that Wnt-β-cat signaling was among the top five enriched terms when analyzing downregulated genes for PMA, ING, and compound **2** (*SI Appendix*, Fig. S8 *J*–*L*).

Notably, however, neither PMA, ING, nor other commercially available lathyrane natural products scored in our β-cat^S37C^-DCK* positive selection assay at any concentration tested, likely due to their toxicity (*SI Appendix*, Fig. S8 *M*–*P*). PMA and ING activate both classical and novel PKCs. Bisindolylmaleimide-I (Bis-I), which inhibits both classical PKCs and novel PKCs, but not Go6976, which only blocks the former, reversed the effects of ING with respect to β-cat, JunB, and Axin 2 levels in the 293FT TK1 KO β-cat^S37C^-DCK* reporter cells and in 293FT cells treated with CHIR99021 (Fig. 4*D* and *SI Appendix*, Fig. S9*A*). Moreover, Bis-I, but not Go6976, blocked the relocalization of endogenous β-cat by ING and compound **2** in DLD-1 cells (Fig. 4*E* and *SI Appendix*, Fig. S9*B*). These observations suggested that β-cat inactivators, such as compound **2**, do so by activating a novel PKC. Consistent with this idea, doxycycline (DOX)-induced expression of constitutively active versions of either PKCδ or PKCθ, but not their catalytically inactive counterparts, phenocopied compound **2**’s effects on both β-cat localization and Axin 2 expression in 293FT cells treated with CHIR99021 (*SI Appendix*, Fig. S9 *C*–*F*). ING still induced the formation of the β-cat cytoplasmic vacuoles in DLD-1 cells in which the classical PKCα was genetically inactivated by CRISPR/Cas9 editing (*SI Appendix*, Fig. S9 *G* and *H*).

Activation of PKC family members characteristically downregulates their apparent abundance in immunoblot assays, due to their decreased stability (42) or reduced immunoreactivity resulting from epitope phosphorylation (43). 293FT cells express multiple PKCs, although PKCα appears to be their primary classical PKC based on protein expression levels [*SI Appendix*, Fig. S9*I*) and (44, 45)]. As expected, PMA and ING activated PKCα and all four novel PKC family members in this assay (Fig. 4*F* and *SI Appendix*, Fig. S9*J*). In contrast, the alcohol-containing **2**, but not the acetylated analogue (**3**), specifically activated the novel PKCs while sparing PKCα.

Compound **2**, but not compound **3**, bound directly to full-length human PKCδ, as determined by microscale thermophoresis, and bound to PKCδ with at least 20× higher affinity than to full-length PKCα (Fig. 4*G* and *SI Appendix*, Fig. S10 *A* and *B*). As expected, PMA and ING bound to both (Fig. 4*G* and *SI Appendix*, Fig. S10 *A* and *B*). Due to the limited supply of compound **2**, these assays were not repeated in the presence of lipids, which are known to enhance the binding of PMA and ING to C1b domains further (46).

Molecular modeling studies suggested that the unique position of the primary alcohol found in compound **2** (on C19, Fig. 3*A*) could adopt a similar hydrogen bond network as known PKC agonists (Fig. 4 *H*–*L*) when docked into the C1b domain of PKCδ (PDB: 7LF3, rat PKCδ, ~98% conserved with human PKCδ). Molecular dynamic simulations indicated that this hydrogen bond network was stable (*SI Appendix*, Fig. S10 *C* and *D*). In sum, the simulations predict a binding mode for compound **2** that traverses the established hydrophobic binding cleft in the C1b domain, a stabilized hydrogen bonding network (Thr242, Leu251, and Gly253), and interaction and stabilization of the active “up” configuration for the novel PKC-specific, diacylglycerol-toggling residue Trp252 (Fig. 4 *H* and *I* and *SI Appendix*, Fig. S10 *C* and *D*) (46). This model also predicts that the acetylated compound **3** would not bind due to steric incompatibility with the shallow binding site and a lack of a free primary alcohol hydrogen bonding. This in silico model of compound **2** binding utilizes the crystal structure of only the C1b domain, which is highly conserved among classical and novel PKCs’ C1 domains, and thus does not take into account the rest of the protein structure that likely defines activity in cells (47, 48).

Finally, we obtained two synthetic PKC agonists, AJH-836 and YSE-028, that specifically activate the novel PKCs and not classical PKCs (49, 50). Both of these compounds are members of the diacylglycerol (DAG) lactone class and bind to the PKC C1 domains (49, 50). As expected, they bound selectively to PKCδ compared to PKCα (*SI Appendix*, Fig. S10 *E* and *F*). Moreover, they phenocopied compound **2** with respect to specific downregulation of novel PKC family members, JunB induction, and β-cat relocalization (*SI Appendix*, Fig. S10 *G* and *H*). Importantly, however, they, like PMA and ING (*SI Appendix*, Fig. S8 *M* and *N*), did not score in our positive selection assay (*SI Appendix*, Fig. S10 *I* and *J*).

## Discussion

PKC activation was historically viewed to be oncogenic, dating back to classical studies with phorbol esters. Nonetheless, there is increasing evidence, including somatic inactivating mutations in cancers and functional studies, to support PKCs as potential tumor suppressors (42). The PKC agonist tool compounds PMA and ING have been reported to have anticancer properties, including in colorectal cancer cells (51), and, like compound **2**, inhibit β-cat function (36, 52). However, PMA and ING appear to be more pan-toxic than **2**, likely in part because they, unlike **2,** also activate classical PKCs. Nonetheless, even DAG lactones that spare classical PKCs failed in our 5-d positive selection assay, perhaps due to off-target effects (53–55) or, in the case of YSE-028, susceptibility to cellular esterases (50). Compound **2** would therefore be a better starting point for developing PKC agonists for cancer therapy.

Additional studies are needed to fully elucidate how compound **2** activates novel PKCs and alters β-cat localization and activity. In perhaps the simplest model, relocalization of β-cat to cytoplasmic vacuoles prevents it from entering the nucleus and thus its ability to directly regulate transcription. We do not understand, however, why this PKC-mediated relocalization also inactivates the DCK* moiety in the β-cat^S37C^-DCK* fusion. Further work is needed to determine whether the inactivation of β-cat and DCK* (in the context of β-cat^S37C^-DCK*) by PKC can be dissociated from their cytoplasmic relocalization by PKC and to elucidate the underlying biochemistry and cell biology responsible for these phenotypes.

Our screen illustrates the complementarity between chemical and genetic screens since the latter typically rely on gene inactivation or quantitative changes in gene expression and hence would not phenocopy PKC activation because activation of PKC requires that it undergo allosteric changes that prevent its autoinhibition (42). Indeed, we confirmed that overexpression of neither wild-type PKCδ nor PKCθ relocalized β-cat, in contrast to their constitutively activate variants. It will be informative to determine the breadth of mechanisms responsible for the different β-cat inactivators in our screen. In this regard, it will be important to retest some of the compounds that did not validate in our initial immunoblot assays, exploring different concentrations and timepoints given the 5-d time course of our screen.

In recent decades the pharmaceutical industry has favored target-based in vitro chemical screens over cell-based phenotypic chemical screens, largely because it can be difficult to identify the targets of hits emerging from the latter. Nonetheless, cell-based screens offer numerous potential advantages, including the ability to discover new biology, interrogate targets in their native contexts, and prioritize compounds that are bioavailable. Although our mechanistic insights related to compound **2** were hypothesis-based, many powerful biochemical (e.g., based on affinity capture) and genetic approaches (e.g., based on the generation of drug-resistant mutants) have been developed to identify targets for compounds scoring in phenotypic screens (56, 57).

Similarly, screening natural product mixtures has largely been abandoned by the pharmaceutical industry (21, 23), despite the fact that natural products have remarkable structural diversity and were the source of many important drugs in the past. Moreover, natural products often have superior absorption, distribution, metabolism, and excretion (ADME) properties compared to the compounds found in most synthetic compound libraries. The use of prefractionated natural product mixtures helps mitigate two main concerns regarding natural product screens: confounding effects caused by toxic chemicals in extracts and difficulties isolating the responsible compounds from extracts that score positively (21, 23). A major problem that remains relates to the ability to resupply extracts in sufficient quantities for purification and downstream chemical and biological analyses. Resupply is particularly important for molecules, such as compound **2**, that cannot be readily synthesized due to their structural complexity. These problems might be partially mitigated by using natural product collections derived from organisms (e.g., bacteria, fungi, certain plants) that can be easily cultured or cultivated at scale. The use of organisms with biosynthetic gene clusters amenable to genetic manipulation could enable the rapid generation of knockout strains for validation purposes and overproducing strains to enhance yields (58).

Drug development success at least doubles when based on a genetically validated target (59, 60). Somatic, and rarely germline, mutations have validated many otherwise undruggable targets in cancer. Cancers are often “addicted” to oncogenic proteins compared to normal cells, even when those proteins are active in the latter, providing a basis for a therapeutic window. Nonetheless, drugging the undruggable and finding drugs with broad therapeutic windows remains a challenge. Both challenges might be aided by screening chemical matter, including natural products, using positive selection assays analogous to the one described here.

## Materials and Methods

### Cell Culture.

HEK293FT (293FT), DLD-1, SNU398, and RKO cells were originally obtained from American Type Culture Collection (ATCC). 293FT TK1 KO cells were generated by CRISPR-Cas9 editing (see below). 293FT, DLD-1, and RKO cells were grown in DMEM (Gibco, 11995065) supplemented with 10% FBS (GeminiBio, 100-106 or, for experiments involving the addition of doxycycline, GeminiBio, 100-800, which is tetracycline free) and 1% penicillin-streptomycin (Gibco, 15140122). SNU398 cells were grown in RPMI-1640 media (Gibco, 11875093) supplemented with 10% FBS (GeminiBio, 100-106; heat inactivated prior to use) and 1% penicillin-streptomycin (Gibco, 15140122). All cell lines were cultured at 37 °C with 5% CO_2_. Lentiviral infected cells were maintained in their corresponding media plus the desired selection agent (blasticidin: 10 to 12.5 µg/mL, puromycin: 2 to 4 µg/mL, or hygromycin: 200 to 300 µg/mL). Cell lines were occasionally tested for *Mycoplasma* infection using MycoAlert *Mycoplasma* Detection Kit (Lonza, LT07-318) or MycoStrip (InvivoGen, rep-mysnc-100).

### Chemicals.

The following compounds were purchased: DMSO (Sigma Aldrich, D2650), thymidine (dThD, MedChemExpress, HY-N1150), (*E*)-5-(2-Bromovinyl)-2’-deoxyuridine (BVdU, Fisher Scientific 50-536-044 from Chem-Impex International Inc. MFCD00058585), CHIR99021 (MedChemExpress, HY-10182), Ingenol-3-angelate (ING, Sigma Aldrich, SML1318), Phorbol-12-myristate (PMA, VWR, 80055-400), Bisindolylmaleimide-I (Bis-I, VWR, 80055-904 and MedChemExpress, HY-13867), Go6976 (Selleck, S7119), Euphorbia factor L1 (Selleck, S9410), Euphorbia factor L2 (MedChemExpress, HY-N5001), Euphorbia factor L3 (MedChemExpress, HY-N0562), Euphorbia factor L7A (MedChemExpress, HY-N8121), Euphorbia factor L7B (MedChemExpress, HY-N8118), 17-AAG (MedChemExpress, HY-10211), Actinomycin D (Thermo Fisher Scientific, 11805017), BIX-02565 (MedChemExpress, HY-16104), WNTinib (MedChemExpress, HY-160765), AZ-628 (MedChemExpress, HY-11004), doxycycline (Takara Bio, 631311), AJH-836 (from the Kazanietz-Cooke Laboratory University of Miami), YSE-028 (a gift from Hirokazu Tamamura, Department of Medicinal Chemistry, Laboratory for Biomaterials and Bioengineering, Institute of Integrated Research, Institute of Science Tokyo, Japan), Zotatifin (gift from Neal Rosen, Emeritus Faculty at Memorial Sloan Kettering Cancer Center, New York, New York), DAPI (Cell Signaling, 4083), Blasticidin (Thermo Fisher Scientific, NC9016621), Puromycin (Thermo Fisher Scientific, NC9138068), hygromycin (Life Technologies, 10687010).

### Immunoblotting.

Cell pellets were lysed in 1× RIPA buffer (Life Technologies, 89901) supplemented with protease inhibitor cocktail (Roche, 11836170001) and phosphatase inhibitor cocktail (Roche, 4906845001). Cell lysates were vortexed for 10 s, incubated on ice for 15 min, vortexed for 10 s again, and then clarified via centrifugation (16,100× g) for 15 min at 4 °C. The supernatants were then transferred to prechilled tubes and the protein concentrations were quantified using the Bradford protein assay (Bio-Rad, 5000006) according to the manufacturer’s protocol. Equalized protein samples were then mixed with Laemmli SDS-Sample Buffer (Boston BioProducts, Inc., 6×-reducing, BP-111R) to a 1× concentration and heated to 70 °C for 10 min. Samples were then either frozen at −80 °C for later use or directly loaded and resolved on Novex Wedge Well protein gels (i.e., 4 to 20% or 4 to 12% Novex WedgeWell, XP04205 or XP04125) using SDS running buffer diluted to 1× working concentration in water [10× SDS running buffer: 29 g Tris-Base (Thermo Fisher Scientific, BP1521), 144 g Glycine (Thermo Fisher Scientific, AA43497A5), 10 g SDS (sodium dodecyl sulfate, Sigma Aldrich, L3771) in 1,000 mL ultrapure water]. Gels were then transferred to nitrocellulose membranes using the Bio-Rad Trans-Blot Turbo Transfer System (Bio-Rad, 1704155 and Bio-Rad, 1704271) with the HIGH MW settings adjusted to achieve a 12 to 14-min transfer time. For some immunoblots, especially when a high molecular weight protein was being compared to a low molecular weight protein (e.g., Fig. 1*D*), a wet PVDF transfer protocol was used instead wherein gels were transferred to PVDF 0.45 µm pore Immobilon Transfer Membranes (Millipore Sigma, IPVH00010) using a Mini-Trans-Blot electrophoresis system (Bio-Rad, 1703930 and Bio-Rad, 1703932). Transfers were done in precooled 1× transfer buffer [400 mL of methanol (Sigma Aldrich, 34860), 28.8 g glycine (Thermo Fisher Scientific, AA43497A5) 12.1 g Tris Base (Thermo Fisher Scientific, BP1521), 1,600 mL of ultrapure water, pH = 8.3] on ice or with a frozen cool pack inside the tank for 2 h at 54 V. For most experiments uniform transfer was confirmed using a brief Ponceau Stain (Cell Signaling Technology, 59803) followed by TBST [6.06 g Tris-Base (Thermo Fisher Scientific, BP1521), 17.5 g sodium chloride (Thermo Fisher Scientific, BP35810) 2 mL Tween-20 (Thermo Fisher Scientific, BP337500), pH to 7.6 via addition of hydrochloric acid (37%, Sigma Aldrich, 258148) washing to remove the stain. Membranes were then blocked with bovine serum albumin (2 g in 40 mL of TBST (see above), Gold BioTechnology, A-420-500] for at least 1 h at room temperature. Primary antibody was then added, and the membranes were incubated overnight at 4 °C on a platform rocker. Membranes were then washed three times with TBST (10 to 20 min per wash) followed by incubation for at least 1 h with the corresponding secondary antibody [horseradish peroxidase (HRP)-conjugated antibody] in TBST. After incubation (room temperature on a platform rocker), membranes were washed at least three times with TBST (20 min per wash). Membranes were then incubated in SuperSignal West Pico Plus solutions (1:1, Thermo Scientific, 1863099 and 1863098). Membranes were then chemiluminescent and colorimetric imaged with a Bio-Rad ChemiDoc MP Imaging system (Version 3.0.1.14) and images contrast/brightness/rotation were uniformly adjusted with High/Low/Gamma settings within the Image Lab 6.1 software (Bio-Rad SOFT-LIT-170-9690-ILSMAC-V-6-1). For all immunoblot membranes, equal loading was confirmed by blotting for a loading control and/or by Ponceau staining. For some composite figures, different loading controls were used for different panels within the figure because of overlap between the molecular weight of the protein of interest and a loading control protein. For example, in Fig. 2 *B*, *D*, and *F*, β-cat and β-actin were from the same membrane and Axin 2 and ERK1/2 were from the same membrane. Not all the loading controls were included in some figures (e.g., Fig. 4*F*) for space and aesthetic reasons. Some images were also previously taken using the Azure biosystem C600 using the instrument software to get chemiluminescent and colorimetric images.

### Antibodies.

The following antibodies were used: anti-TK1 (Cell Signaling Technology, 8960), anti-V5-HRP (Life Technologies, R96125, 1:9,000), anti-β-Actin-HRP (Cell Signaling Technology, 5125S, 1:5,000), anti-Axin2 (Cell Signaling Technology, 2151), anti-β-Cat unP-S45 (Cell Signaling Technology, 19807S, 1:2,000), anti-β-Cat unP-S33/S37/T41 (Cell Signaling Technology, 8814S, 1:2,000), anti-β-Cat total (Cell Signaling Technology, 8480, 1:2,000), anti-β-Cat total (Santa Cruz Biotechnology, SC-7963), anti-ERK1/2 (Cell Signaling Technology, 4695), anti-JunB (Cell Signaling Technology, 3753), anti-GAPDH (Cell Signaling Technology, 5174), anti-PKCα (Cell Signaling Technology, 59754), anti-PKCβ (Cell Signaling Technology, 46809), anti-PKCδ (Cell Signaling Technology, 2058), anti-PKCε (Cell Signaling Technology, 2683), anti-PKCη (Abcam, ab179524), anti-PKCθ (Cell Signaling Technology, 13643), anti-Phospho-(Ser) PKC Substrate (Cell Signaling Technology, 2261), anti-Phospho-PKC (pan) (beta II Ser660) (Cell Signaling Technology, 9371), anti-PKCδ (phosphor S299) (Abcam, AB133456), anti-DCK (Abcam, ab151966), anti-GFP (Cell Signaling Technology, 2956), anti-FLAG (Cell Signaling Technology, 14793), anti-vinculin (Sigma-Aldrich, V9131), anti-rabbit-HRP (Thermo Fisher Scientific, NC9611376, secondary antibody), anti-mouse-HRP (Thermo Fisher Scientific, NC9491974, secondary antibody), anti-mouse-AlexaFluor Plus 555 (Life technologies, A32727, 1:500), anti-rabbit-AlexaFluor Plus 488 (Life Technologies, A32731, 1:500). All primary antibodies were used at a 1:1,000 dilution unless otherwise stated. All secondary antibodies were used at a 1:10,000 unless otherwise stated.

### Plasmids.

Plasmids were generated as described in *SI Appendix, Materials and Methods*.

### BVdU High-Throughput Screening (HTS) Assay.

293FT TK1 KO full-length β-cat^S37C^-DCK*, C-terminal truncated ^1-665^β-cat^S37C^-DCK*, full-length β-cat^S37C/S715A^-DCK*, DCK*-IKZF1 or DCK* cells were seeded into 384-well plates (Corning, 3764) at 450, 450, 450, 300, or 200 cells/well respectively in 30 µL of media via MultiDrop Combi (Thermo Scientific, 5840330) dispensing. Plates were then centrifuged at 218× g for 10 s and cells were allowed to adhere overnight. The next morning, the plates were treated with test samples (e.g., natural product fractions) via acoustic dosing using an Echo 655 liquid handler. Positive (dThD) (column 2) and negative (DMSO) (column 1) controls were then dosed via Hewlett Packard (HP) D300e (Tecan) dispensing, normalized to the amount of DMSO dispensed during the acoustic dispensing phase. In some experiments, a dose response of different compounds of interest were dosed via HP D300e dispensing at this point. Plates were then centrifuged at 218× g for 10 s and incubated. The next day, 10 µL of BVdU media stock solution was added to each well using a MultiDrop Combi Dispenser (Thermo Scientific, 5840330). The concentration of the BVdU media stock solution was calculated to achieve the desired final BVdU concentration (10 µM for DCK*, 50 µM for β-cat^S37C^-DCK*, 50 µM for C-terminal truncated β-cat^S37C^-DCK*, 100 µM for DCK*-IKZF1). Plates were then centrifuged at 218× g for 10 s and incubated for 96 h. Note: Since there is a significant dilution after BVdU media stock solution addition (30 µL to 40 µL), we chose to always refer to the final concentration at day 2 for a given test sample or control even though from day 1 to day 2 of the assay there is a 1.3× concentration as compared to the final concentration. The GFP fluorescence of each well was then quantified using an Acumen eX3/HCI (STP LabTech) laser scanning cytometer. GFP fluorescence was quantified by defining the metric “GFP+ Object” to identify GFP+ cells while excluding general debris.

### Natural Product HTS.

Using the BVdU HTS assay, 326,304 natural product samples were tested using a Beckmann Echo 655 for dispensing (100 nL of 2.5 mg/mL stock). Extracts and fractions were tested at a final concentration of 6.25 µg/mL. After testing, GFP+ object values were converted to a Z-score (based on the variation of experimental values for a given assay plate). A hit was defined as any fraction with a Z score ≥ 3 in β-cat^S37C^-DCK* cells and a Z score < 1 in DCK* cells. A small number of fractions that just missed the hit metric criteria were included. This yielded 736 hit fractions that were the basis for cherry pick reconfirmation assay (see below).

### Subfraction Secondary Screening.

Using the standard BVdU HTS assay, subfractions were tested at two doses: 100 nL (3.3 µg/mL day 1, 2.5 µg/mL at day 2 to 6) and 200 nL (6.7 µg/mL day 1, 5.0 µg/mL at day 2 to 6). GFP+ object values were then converted to a relative GFP+ object value (fold protection) by dividing that number by the number of GFP+ objects observed for averaged negative control wells. A hit subfraction was defined as having a fold protection ≥3 in β-cat^S37C^-DCK* cells, ≤1.2 in DCK*, and ≤1.5 in DCK*-IKZF1 at any dose tested.

### Immunofluorescence.

DLD-1 cells were seeded in 8-well chambered plates (Ibidi, 80826, 1 × 10^4^ cells in 300 µL of media per well). The next morning, the cells were dosed with the indicated drug in diluted media stocks (50 µL, final DMSO %: 0.5%). 24 h later, the media were removed and the cells were washed with DPBS (~300 µL, Gibco, 14190094), then fixed with 4% paraformaldehyde (PFA, Life Technologies, J61899.AK; 30-min incubation). The cells were then washed two times with DPBS and then blocked and permeabilized with a 5% BSA (Gold BioTechnology, A-420-500) and 0.3%TritonX-100 (Sigma Aldrich, 648463) in DPBS solution (2 g BSA, 1.2 mL 10% Triton X-100 in 38.8 mL DPBS). The cells were incubated for at least 1 h, washed three times with DPBS, then incubated with desired primary antibodies (1:500, e.g., anti-β-Cat total Santa Cruz Biotechnology, SC-7963) in 5% BSA in DPBS (2 g BSA in 40 mL DPBS) at 4 °C for at least 16 h. The next morning, the cells were washed three times with DPBS (~300 µL) and then incubated with the desired secondary antibody [1:500, e.g., anti-mouse-AlexaFluor Plus 555 (Life technologies, A32727) or anti-rabbit-AlexaFluor Plus 488 (Life Technologies, A32731)] for 2 h at room temperature. The cells were then washed two times with DPBS, incubated with a DAPI in DPBS solution for 5 min [1 µL of DAPI solution (Cell Signaling, 4083) in 8 mL of DPBS], washed again two times with DPBS, and finally left in 300 µL of DPBS. The wells were then imaged on a Nikon Eclipse Ti2 confocal superresolution microscope (NIS Elements Version 5.42.01 software). Conventional W1 spinning disk images and SORA enhanced images were taken for wells with a 60× oil immersion objective lens and 405 nm or 561 nm lasers. For each individual experiment, the same exposure times and laser powers was utilized for all wells. Images were then processed using ImageJ (Fuji) and brightness and contrast settings were normalized across the experiment. A single Z plane is shown for images herein even though in some instances a full Z-stack was obtained (20 to 40 images at 0.25 µm steps).

For the experiments involving DOX-inducible expression of PKC isoforms, the DLD-1 cells were seeded in 8-well chambered plates (Ibidi, 80826, 3 × 10^4^ cells in 250 µL of tetracycline negative media per well). The next morning the cells were treated with doxycycline (DOX, Takara Bio, 631311) for 6 h (added as a diluted, tetracycline negative-media stock to desired final concentration of 100 ng/µL). Immunofluorescence assays were then performed as described above.

### Additional Experimental Details.

Further experimental details, including chemistry, natural product isolation, and biological assessments, are described in *SI Appendix, Materials and Methods*.

## Supplementary Material

Appendix 01 (PDF)

Dataset S01 (XLSX)

Dataset S02 (XLSX)

## Data Availability

RNAseq data were deposited in the public functional genomics data repository GEO; accession number GSE308354 (61). Experimental high-resolution LC–MS and NMR data for compounds **2** and **3** and computational data for optimized conformers for NMR and ECD spectral calculations for compounds **2** and **3** have been deposited in the Harvard Dataverse (62). The primary HTS data are available at NCI Program for Natural Products Discovery (NPNPD) Public Data Wiki (63). All other data are included in the article and/or supporting information.
